# Psychological factors associated with bruxism in a sample of university students from the Colombian Caribbean

**DOI:** 10.1192/j.eurpsy.2023.1434

**Published:** 2023-07-19

**Authors:** K. Cabas-Hoyos, A. Llinas-Ariza, C. Guerrero-Cantillo

**Affiliations:** 1 Cognición y Eduación; 2 Program of Odontology; 3 Universidad del Magdalena, SANTA MARTA, Colombia

## Abstract

**Introduction:**

Within the theories that explain tooth grinding are dental factors, skeletal malocclusions, occlusal anomalies and defective reconstructions, however, there would be psychological factors that explain this phenomenon.

**Objectives:**

The aim of the study is to evaluate anxiety, depression and coping responses in university students who self-report clenching or grinding teeth.

**Methods:**

Design: Non-experimental, cross-sectional, and quantitative.

Sample: University students (n=25) aged between 18-25 years (mean: 25.3; SD: 2.39), purposive sampling. Participants completed a self-reported questionnaire reporting teeth clenching/grinding habits.

Instruments: Standardized instruments were used, such as the State Trait Anxiety Inventory (STAI), Beck’s Depression Inventory (BDI) and Coping Strategies Scale Modified (CSS-M).

Procedure: First, the study was disclosed, the participants were informed and signed informed consent. They were asked to complete an instrument on teeth grinding/clenching habits and if it was fulfilled, it was included in the study and the psychological evaluation instruments were administered.

Data analysis: An analysis was made using descriptive statistics.

**Results:**

The STAI results showed a high Anxiety-State in all the participants and the Anxiety-Trait had a prevalence of 92.3%. Regarding the levels of depression, it was evidenced that 7.7% presented moderate depression and 31.6% showed mild symptoms.

The most used coping strategies were problem solving (87.2%), positive reappraisal (74.4%) and religious support (71.8%), while the least used were seeking professional help (92.3%), waiting (76.9%), aggressive reaction (74.4%) and expression of coping difficulty (71.8%).

**Conclusions:**

University students must cope with an academic load that exceeds their capacity to face academic challenges (Wikes et al., 2019). This demand causes significant discomfort that increases emotions with a high negative charge and favors the appearance or intensification of mental health problems, such as chronic stress, anxiety, depression, nervousness and behavioral disorders.

**Disclosure of Interest:**

None Declared

